# Abnormal brain synchrony in Down Syndrome^☆^

**DOI:** 10.1016/j.nicl.2013.05.006

**Published:** 2013-05-24

**Authors:** Jeffrey S. Anderson, Jared A. Nielsen, Michael A. Ferguson, Melissa C. Burback, Elizabeth T. Cox, Li Dai, Guido Gerig, Jamie O. Edgin, Julie R. Korenberg

**Affiliations:** a Division of Neuroradiology, University of Utah, USA; b Interdepartmental Program in Neuroscience, University of Utah, USA; c The Brain Institute at the University of Utah, USA; d Department of Bioengineering, University of Utah, USA; e Department of Pediatrics, University of Utah, USA; f Scientific Computing and Imaging Institute, University of Utah, USA; g Department of Psychology University of Arizona

## Abstract

Down Syndrome is the most common genetic cause for intellectual disability, yet the pathophysiology of cognitive impairment in Down Syndrome is unknown. We compared fMRI scans of 15 individuals with Down Syndrome to 14 typically developing control subjects while they viewed 50 min of cartoon video clips. There was widespread increased synchrony between brain regions, with only a small subset of strong, distant connections showing underconnectivity in Down Syndrome. Brain regions showing negative correlations were less anticorrelated and were among the most strongly affected connections in the brain. Increased correlation was observed between all of the distributed brain networks studied, with the strongest internetwork correlation in subjects with the lowest performance IQ. A functional parcellation of the brain showed simplified network structure in Down Syndrome organized by local connectivity. Despite increased interregional synchrony, intersubject correlation to the cartoon stimuli was lower in Down Syndrome, indicating that increased synchrony had a temporal pattern that was not in response to environmental stimuli, but idiosyncratic to each Down Syndrome subject. Short-range, increased synchrony was not observed in a comparison sample of 447 autism vs. 517 control subjects from the Autism Brain Imaging Exchange (ABIDE) collection of resting state fMRI data, and increased internetwork synchrony was only observed between the default mode and attentional networks in autism. These findings suggest immature development of connectivity in Down Syndrome with impaired ability to integrate information from distant brain regions into coherent distributed networks.

## Highlights

• Adjacent brain regions are more synchronized in Down Syndrome.
• Distant brain regions show less synchronization in Down Syndrome.
• Negatively correlated brain regions are less anticorrelated in Down Syndrome.
• Down Syndrome subjects show simplified brain network architecture.
• Increased brain synchrony does not reflect a response to environmental stimuli.

## 1 Introduction

Down Syndrome (trisomy 21) is the most common genetic cause of intellectual disabilities. Down Syndrome occurs in 9.0 to 11.8 per 10,000 live births (Shin et al., 2009), and is associated with impairments in language (Martin et al., 2009), cognition (Silverman, 2007), learning and memory (Jarrold et al., 2000). Although clinical features of Down Syndrome and the DNA sequence of chromosome 21 have been characterized, few neuroimaging studies have characterized the pathophysiology of neurological deficits in Down Syndrome. Early structural MRI reports suggested that total intracranial volume is smaller in Down Syndrome, with the greatest volumetric differences in the cerebellum, brainstem, and frontal lobes (Aylward et al., 1999; Kesslak et al., 1994; Raz et al., 1995). Even after correction for total brain volume, hippocampal volumes have been found to be smaller than for typically developing individuals (Pinter et al., 2001). A voxel-based morphometric analysis similarly showed decreased volume in the brainstem, cerebellum, cingulate gyrus, medial frontal lobe, superior temporal lobes, and hippocampi (White et al., 2003), with an independent study subsequently showing reduced gray matter in the inferior cerebellum, fusiform gyrus, and medial temporal lobe (Menghini et al., 2011). Given that adults with Down Syndrome show accelerated volume loss, even in the absence of dementia (Beacher et al., 2010; Haier et al., 2008; Teipel et al., 2004), these findings may suggest a pattern of volume loss in brain regions relevant to the markedly accelerated onset of Alzheimer Disease in Down Syndrome (Mann, 1988). Nevertheless, beyond consistently reduced total intracranial volume, structural MRI studies have been heterogeneous in their characterization of specific brain regions showing decreased volumes.

Less is known about the functional architecture of the brain in Down Syndrome. Magnetic resonance spectroscopic differences include an elevated myoinositol peak in subjects with Down Syndrome, with (Lamar et al., 2011) or without (Beacher et al., 2005) dementia, but without changes in creatine or N-acetylaspartate (Shonk and Ross, 1995). An activation study using magnetoencephalography (MEG) indicated that different subjects with Down Syndrome may differ in the ipsilateral vs. contralateral response to a finger movement task (Virji-Babul et al., 2011). Electroencephalographic analysis has shown decreased amplitude of synchronized alpha rhythms, suggesting impaired cortical neural synchronization (Babiloni et al., 2010). A study of fMRI activation during passive story listening showed decreased activation in classical receptive language areas in Down syndrome compared with controls (Losin et al., 2009). A subsequent study during an object recognition task showed different associations between activation and visuo-spatial ability in Down Syndrome and typically developing controls (Jacola et al., 2011). While these studies suggest altered brain metabolism and activation patterns in Down Syndrome, no studies to date have been reported using functional connectivity MRI or diffusion tensor MRI techniques in Down Syndrome.

Functional MRI connectivity (fcMRI) has developed from the observation that functionally related brain regions exhibit temporally synchronized fluctuations in blood oxygen level dependent (BOLD) signal (Biswal et al., 1995). By measuring the temporal correlation between brain regions, fcMRI studies have revealed a functional network anatomy comprised of numerous distributed brain networks associated with distinct functional domains (Power et al., 2011; Yeo et al., 2011). Among these networks are spatially heterogeneous ensembles of brain regions that correspond with high-level cognitive perception. An attention control network (also referred to as the task positive network or executive network) processes attention to external stimuli (Anderson et al., 2010; Corbetta and Shulman, 2002; Fox et al., 2006). Attention to internal stimuli or narrative is processed by the brain's default mode network, also referred to as the task negative network (Raichle and Snyder, 2007; Raichle et al., 2001). Processing of novel stimuli is performed by the salience network, or cingulo-insular network (Seeley et al., 2007). Additional networks are associated with sensorimotor, visual, auditory, and language function (Damoiseaux et al., 2006).

High-level distributed brain networks requiring synchronous communication across many brain regions in both hemispheres have been shown to be abnormal in other neurodevelopmental disorders. Reported findings include immaturity of brain network development in Tourette Syndrome (Church et al., 2009), generalized underconnectivity in autism (Anderson et al., 2011d; Just et al., 2004; Muller et al., 2011), abnormal attentional network organization in attention deficit hyperactivity disorder (Castellanos et al., 2008; Fair et al., 2010), and abnormal cinguloinsular connectivity in obsessive compulsive disorder (Cocchi et al., 2011). Given the specificity of connectivity disturbances in other developmental disorders, patterns of abnormal connectivity may constrain hypotheses of neuropathological mechanisms in Down Syndrome.

There are challenges to studying functional connectivity in a Down Syndrome population that is severely low-functioning and prone to high levels of subject motion during scanning, particularly given the relatively noisy functional connectivity measurements in individual subjects (Anderson et al., 2011c; Shehzad et al., 2009). Although longer scan times may improve reliability of measurements for each subject, long scan times result in more subject motion and greater heterogeneity of cognitive state, with high risk of subjects falling asleep during the scan. We approached this problem by scanning 15 Down Syndrome subjects and 14 healthy control subjects for extended time periods (50 minute BOLD imaging per subject) while they watched cartoon stimuli. A previous study has shown smaller test–retest variance of functional connectivity measurements obtained during the same cartoon stimuli than during undirected wakefulness (Anderson et al., 2011c). We evaluated the two groups using a dense array of connections between all gray matter regions, as well as with metrics of inter-network connectivity. By taking advantage of the fixed timing of the stimuli across subjects, we also evaluated for inter-group differences in the time course of regional brain activation. We also compared the findings in Down Syndrome to a large sample of resting state fMRI scans in autism to evaluate whether abnormalities are distinct in different neurodevelopmental disorders.

## 2 Materials and methods

### 2.1 Subject characteristics

All analyses and data collected for this study were performed in accordance with guidelines established by the University of Utah Institutional Review Board and after obtaining informed consent or assent from all Down Syndrome and control participants and consent from all guardians of Down Syndrome participants. Data from anonymized publicly available datasets were all shared in accordance with guidelines established by human subject protection boards of the corresponding institutions as described on the project websites.

Sixteen subjects with Down Syndrome were recruited from the community with genotyping in all cases to confirm trisomy 21. One subject exhibited genetic mosaicism for Down Syndrome, but showed facial, behavioral, and cognitive deficits characteristic of Down Syndrome. One subject with Down Syndrome was excluded from all analyses due to excessive motion during the scan. Control subjects were also recruited from the community. Medical history and structured psychiatric interview were performed. No control subjects had history of developmental, learning, cognitive, neurological, or neuropsychiatric Axis I condition. Verbal IQ (VIQ) and performance IQ (PIQ) were measured with the Kaufman Brief Intelligent Test, Second Edition (Kaufman, 1990). IQ measurements were performed in 6/14 control and 12/15 Down Syndrome subjects. A larger control sample of resting state fMRI scans was used to determine mean correlation for bins of correlation strength and path length. These subjects were derived from the 1000 Functional Connectomes project (FCON 1000, n = 623) and typically developing subjects from the ADHD200 dataset (n = 396). Inclusion criteria for these datasets were ages 7–30, but otherwise as specified in a prior report (Anderson et al., 2011b).

**Fig. 1:**
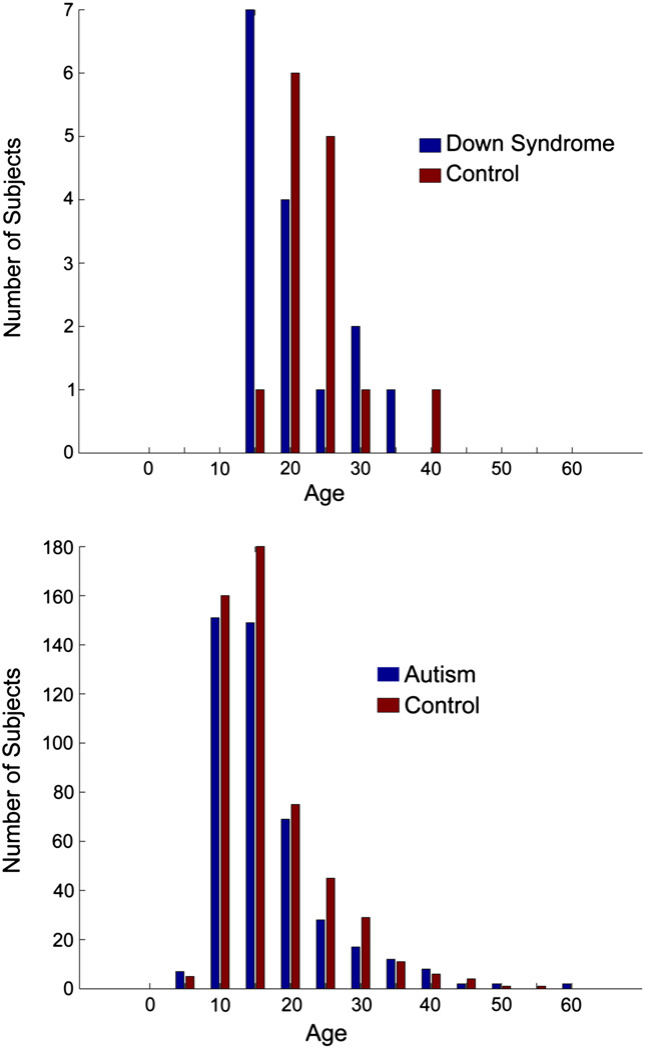
Age distribution of subjects used in the analysis. Down Syndrome and control participants are shown above, with ABIDE sample (both autism and control) shown below. Histograms show age in 5-year bins.

An additional patient and control cohort was included from the Autism Brain Imaging Exchange (ABIDE) dataset consisting of 964 subjects (517 typically developing subjects and 447 subjects with autism) from 16 sites and 19 datasets (DiMartino et al., 2013). Each site followed different criteria for diagnosing patients with autism or ascertaining typical development. Nevertheless, the majority of the sites used the Autism Diagnostic Observation Schedule and Autism Diagnostic Interview—Revised. Specific diagnostic criteria for each site can be found at fcon_1000.projects.nitrc.org/indi/abide/index.html. Subject demographics are shown in Table 1. A histogram of the age distribution is shown in Fig. 1 for Down Syndrome, control, and ABIDE datasets.

### 2.2 Data acquisition

Images were acquired on Siemens 3 Tesla Trio scanner with 12-channel head coil. The scanning protocol consisted of initial 1 mm isotropic MPRAGE acquisition for an anatomic template. BOLD echoplanar images (TR = 2.0 s, TE = 28 ms, GRAPPA parallel acquisition with acceleration factor = 2, 40 slices at 3 mm slice thickness, 64 × 64 matrix) were obtained while viewing Bugs Bunny cartoons (Looney Tunes Golden Collection Volume 1, Warner Home Video) (Anderson et al., 2011c). These consisted of ten five-minute clips, none of which contained a complete cartoon. The following 10 clips were used, beginning at the opening credits for each clip: “Baseball Bugs,” “High Diving Hare,” “Bully for Bugs,” “What's Up Doc,” “Ballot Box Bunny,” “Rabbit of Seville,” “Wabbit Twouble,” “Rabbit's Kin,” “Long-Haired Hare,” and “Rabbit Seasoning.” Both auditory and visual components of the video were presented. Subjectively, both control and Down Syndrome participants appeared to tolerate the cartoons well and remained awake during the stimuli as observed by live video feed of the subjects' eyes during examination. Data was obtained during the 10 five-minute cartoons for each subject. The stimulus computer was synchronized to the onset of the first BOLD image via fiber optic pulse emitted by the scanner for reproducible, precise onset timing.

### 2.3 fMRI preprocessing

Offline preprocessing was performed in MATLAB (Mathworks, Natick, MA) using SPM8 (Wellcome Trust, London) software. Initial slice timing correction was performed to adjust for interleaved slice acquisition. All images were motion corrected using realign procedure. BOLD images were coregistered to the MPRAGE anatomic image sequence for each subject. All images were normalized to the MNI template brain (T1.nii in SPM8), with manual inspection of appropriate normalization in all subjects.

To correct for BOLD signal attributable to physiological noise such as heart rate and respiration, we used a regression algorithm using time series from voxels in the facial soft tissues, CSF and white matter to correct for artifactual correlations in the BOLD data (Fox et al., 2009). No global signal regression was performed, to avoid introducing artifactual anticorrelations in the data (Anderson et al., 2011a; Murphy et al., 2009; Saad et al., 2012). Scalp and facial soft tissues, CSF and white matter signal regression were performed after automated gray matter, white matter, and CSF segmentation of each subject's MPRAGE volume using SPM8. These segmented images were inspected manually to confirm appropriate identification of tissue components. The CSF time series for each subject was measured from the lateral ventricles. This was obtained from selecting voxels from the CSF segmented image for each subject within the bounding box defined by MNI coordinates: − 35 < x < 35, − 60 < y < 30, 0 < z < 30. White matter time series for each subject were obtained from the mean time series of voxels within 2 ROIs in the bilateral centrum semiovale (MNI coordinates: *left*: x = − 27, y = − 7, z = 30; *right*: x = 27, y = − 7, z = 30, each ROI had a 10-mm radius).

Before extracting the white matter time series, an exclusive mask was performed with the gray matter segmented image from each subject to eliminate voxels containing gray matter. A soft tissue mask of the facial and scalp soft tissues was used as previously described (Anderson et al., 2011a). The mean soft tissue, CSF and white matter time series were then used as regressors in a general linear model (glmfit.m in MATLAB Statistics Toolbox) for the BOLD time series at each voxel in the brain, and the best fit was subtracted from the voxel's time series data, producing the signal-corrected time series images. Each voxel's time series was bandpass filtered with a frequency window of 0.001 Hz to 0.1 Hz (Cordes et al., 2001) and linearly detrended to correct for scanner drift. No smoothing was performed. Each frame was then inspected for significant motion using procedure reported by Power et al. (2012), and frames with DVARS or root-mean-square motion parameters > 0.2 were removed prior to analysis of connectivity results.

### 2.4 Network and ROI selection and grouping of ROIs into distance/correlation bins

Analysis of brain networks was performed using a published parcellation of the brain into functional networks by Yeo et al. (2011). We used the 7-network parcellation of the brain as a metric of segmentation and differentiation of distributed brain networks. The mean time series was averaged for all voxels within each of the 7 networks in each subject, and Fisher-transformed correlation was measured for each pair of networks in each subject.

For finer spatial resolution, 7266 ROIs were selected to form a lattice covering the gray matter as previously described (Anderson et al., 2011b, 2011d; Ferguson and Anderson, 2011). The ROIs averaged 4.9 +/− 1.3 s.d. voxels in size for 3 mm isotropic voxels. For each subject, the preprocessed BOLD time series was averaged from the voxels in each of the 7266 ROIs. Pearson correlation coefficients were calculated for each pair of voxels to obtain a 7266 × 7266 correlation matrix (26,393,745 connections per subject), and all correlation values were converted using Fisher Z-transformation (Fox et al., 2009; Kennedy and Courchesne, 2008; Lowe et al., 1998; Murphy et al., 2009). The same processing procedure was also performed for 1019 subjects from the 1000 Functional Connectome and ADHD 200 datasets, previously described (Anderson et al., 2011b; Ferguson and Anderson, 2011), and 964 subjects from the ABIDE dataset. Mean correlation between each pair of ROIs from this dataset was used to group connections into bins based on Euclidean distance between the ROIs and mean correlation from the 1019-subject dataset.

### 2.5 Graph-theoretical analysis

The functional network structure was investigated by first averaging the 7266 × 7266 correlation matrices separately for the Down Syndrome and two healthy control groups. Then, an undirected binary graph with a 7% tie density threshold was calculated. Finally, the Infomap algorithm was implemented to divide the 7266 ROIs into networks or communities (Rosvall and Bergstrom, 2008).

Clustering coefficient is a measure of segregation and is the proportion of an individual ROI's neighbors that share a connection. A 7266 × 7266 binary graph was formed for each subject across a correlation range of absolute thresholds between 0.1 and 0.5. The clustering_coef_bu.m function found at http://www.brain-connectivity-toolbox.net/ was utilized to calculate the clustering coefficient for each of the 7266 ROIs and then averaged across each subject (Rubinov and Sporns, 2010).

### 2.6 Intersubject synchronization

All control and Down Syndrome subjects were scanned while watching the same 10 cartoons. To test the relative timing of brain activation phase locked to these stimuli, an intersubject synchronization analysis was performed (Hasson et al., 2004, 2010). The concatenated time series from the three cartoons for each of the 7266 ROIs was obtained for each subject and correlation coefficient was obtained between homologous ROIs for each control/control, control/Down Syndrome, and Down Syndrome/Down Syndrome pair of subjects. Significant correlation across control/control subject pairs was evaluated with a Wilcoxon rank sum test between correlation of pairs of time series with analogous time series obtained by scrambling the order of one of the time series, with acceptable false discovery rate of q < 0.05 across ROIs.

## 3 Results

fMRI scans were obtained for 16 individuals with Down Syndrome and 14 healthy control individuals (50 minute BOLD imaging per subject, 1550 volumes). Down Syndrome volunteers were more apprehensive about MRI imaging than the control group, and had more difficulty remaining still during the scans. Measurements of subject motion were significantly higher in Down Syndrome subjects than for controls. To mitigate subject motion and facilitate longer scan times with a more homogeneous cognitive state (Anderson et al., 2011c), all BOLD imaging was performed while subjects watched video clips of Bugs Bunny cartoons. We calculated framewise displacement as the sum of the six motion parameters obtained from image realignment and found that mean framewise motion was 0.18 +/− 0.07 mm for Down Syndrome subjects and 0.07 +/− 0.02 mm for healthy controls (p = 0.000016). Data from one Down Syndrome subject were discarded because greater than 50% of frames exhibited > 0.2 mm motion, leaving 15 Down Syndrome subjects in subsequent analyses.

Because even small differences in motion have been associated with differential functional connectivity results (Power et al., 2012; Satterthwaite et al., 2012; Van Dijk et al., 2012; Yan et al., 2013), we performed additional motion scrubbing by removing all volumes immediately before and after a framewise displacement of greater than 0.2 mm, with concatenation of remaining volumes (Power et al., 2012). This resulted in exclusion of an average of 513 +/− 221 volumes (max 740) for Down Syndrome subjects and 74 +/− 62 volumes (max 176) for healthy controls from the 1550 total volumes. After motion scrubbing, Down Syndrome subjects showed mean framewise displacement of 0.091 +/− 0.014 mm and healthy controls showed 0.062 +/− 0.012 mm. Although the difference in motion was markedly reduced after scrubbing, a significant difference persisted, so we also analyzed the Down Syndrome group results allowing only volumes with framewise displacement less than 0.1 mm, which resulted in mean framewise displacement of 0.062 +/− 0.005 mm, identical to the healthy control group (p = 0.68). This condition resulted in exclusion of 723 +/− 240 (max 1188) volumes from the Down Syndrome group. None of the analyses showed appreciable differences in results between the 0.1 and 0.2 threshold scrubbing condition for Down Syndrome, leaving us to conclude we have successfully identified relatively motion-free epochs for analysis, and the 0.2 threshold condition was used for the results below for both Down Syndrome and healthy control groups.

### 3.1 Increased inter-regional synchrony in Down Syndrome

**Fig. 2:**
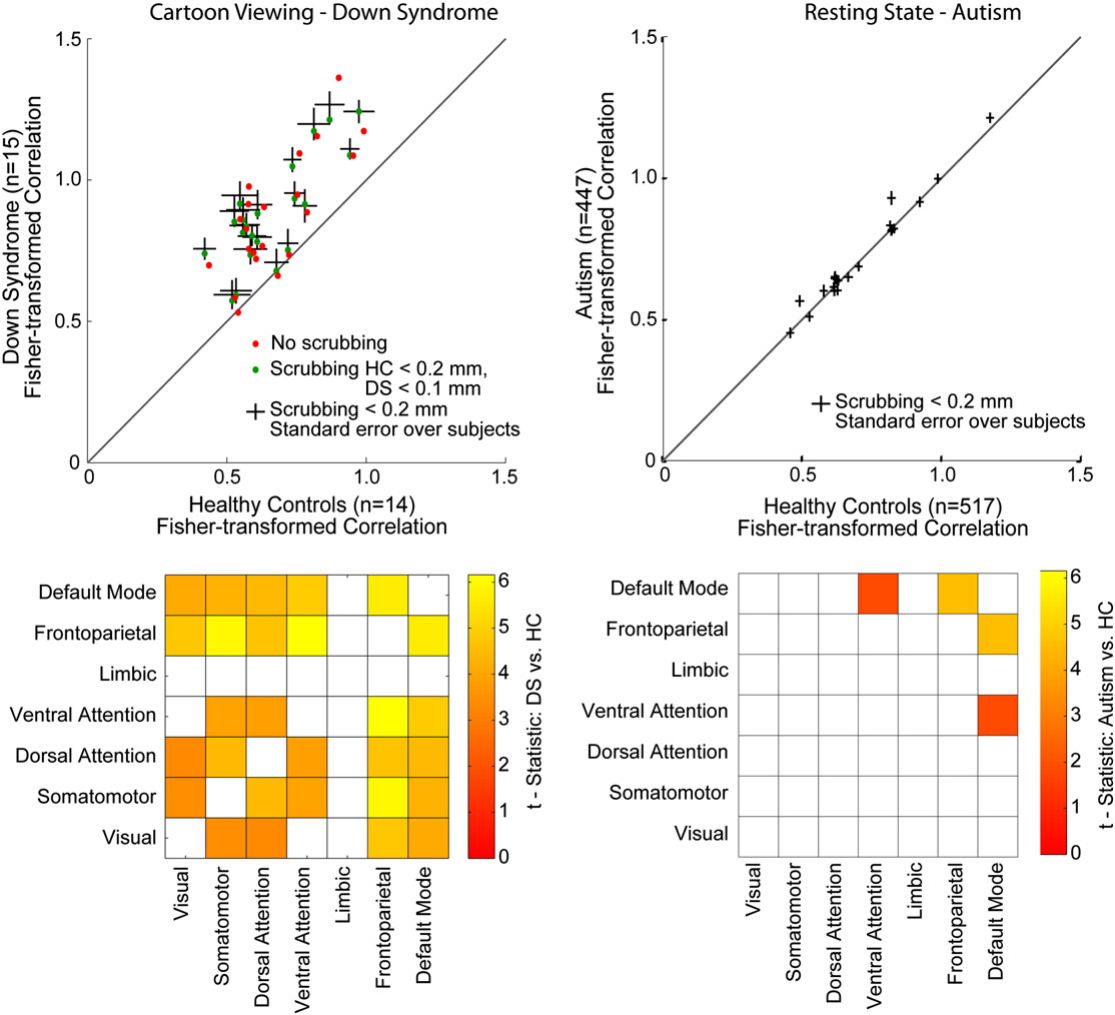
Between-network connectivity differences. Mean time series were obtained from voxels comprising 7 non-overlapping distributed networks in the brain for each subject and Fisher-transformed correlation was measured in each subject between the time series for each pair of networks. Top left; Mean correlation values for control and Down Syndrome groups are shown for each pair of networks. Vertical and horizontal lines show standard error of the mean across subjects for each network pair. The diagonal line shows y = x. Green circles show results for mean inter-network correlation using a 0.1 mm threshold for motion scrubbing in the Down Syndrome group, and red circles show analogous results without any motion scrubbing. Top right; Analogous scatter plot of between-network synchrony is shown for the ABIDE dataset. Vertical and horizontal lines show standard error of the mean across subjects for each network pair. Bottom left; Significant increased (colored) between-network synchrony was found for 14 of 21 network pairs using FDR q < 0.05 across network pairs. T-scores for a two tailed *t*-test for each network pair are shown by the color scale. Bottom right; Significant increased between-network synchrony was observed only for 2 network pairs in the autism sample.

We first evaluated for differences in synchrony between distributed brain networks. The networks were defined a priori by using the 7-network brain parcellation obtained from Yeo et al. (2011). An average time series was obtained for each subject from the voxels comprising each of the 7 networks (after removal of volumes with head motion), and Fisher-transformed correlation was measured between the time series of each pair of networks. Results are shown in Fig. 2. In every case, there was greater synchrony between the networks for Down Syndrome subjects than for control subjects, significant after multiple comparison correction in 14 out of 21 network pairs with acceptable false discovery rate q < 0.05 across the 21 measurements.

The autism cohort showed a different pattern of between-network synchrony abnormalities. Differences were only detected for default mode vs. attentional network pairs (DMN vs. ventral attention network and DMN vs. frontoparietal executive network). Despite a much larger sample size, the abnormalities in autism were much smaller in magnitude than for Down Syndrome, more closely approximating the results in controls. Nevertheless, it is possible that sensitivity for abnormalities in autism is limited due to intersite variability in technique and population. The results may also be influenced by technique, since autism results were acquired during a resting state scan and Down Syndrome results during cartoon viewing.

**Fig. 3:**
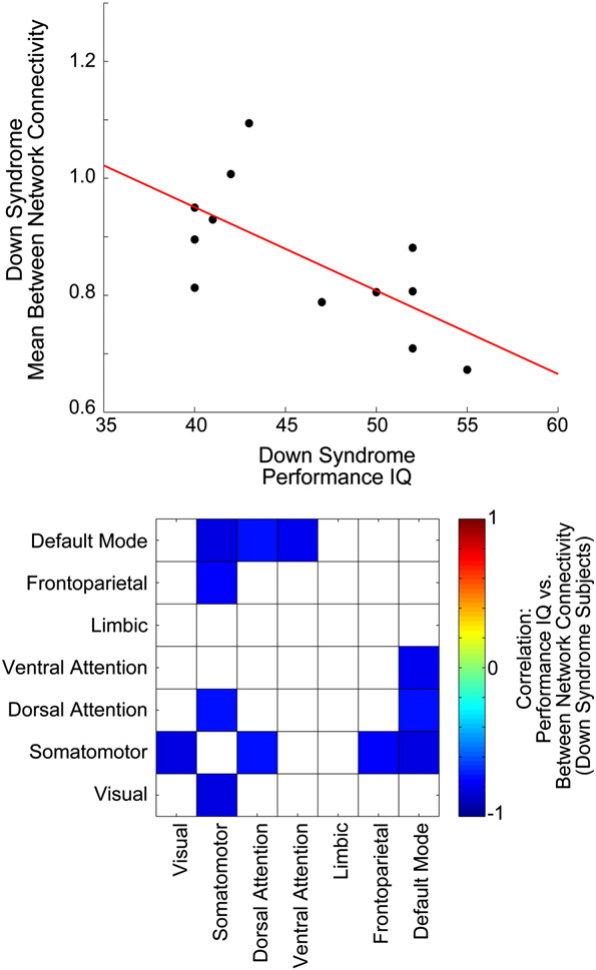
Inverse relationship of internetwork correlation and performance IQ in Down Syndrome. Top; Scatter plot showing mean Fisher-transformed internetwork correlation (averaged across all network pairs) compared to performance IQ for Down Syndrome subjects. Bottom; Significant correlations between individual network pairs and Down Syndrome performance IQ, with acceptable False Discovery Rate q < 0.05 among all network pairs.

Results for both the 0.1 mm and 0.2 mm motion scrubbing conditions are shown in the scatter plot to illustrate that small residual micro-movements had little effect on the results. The results indicate widespread increased synchrony between brain networks in Down Syndrome with less differentiation of the individual networks' temporal activity. Once abnormally increased internetwork correlation was observed, a post-hoc comparison to IQ was performed to evaluate whether this finding covaried with IQ as a measurement of cognitive impairment. Between network synchrony was inversely related to performance IQ in the Down Syndrome subjects (r = − 0.67, p = 0.024, two-tailed *t*-test; Fig. 3). No relationship was seen with internetwork correlation and verbal IQ. Although intriguing, we consider this relationship exploratory given that this was constructed from a subset of an already modest Down Syndrome sample (n = 11).

No relationships were seen with either verbal or performance IQ in autism and between network correlation for any of the 21 network pairs examined. No differences in any of the 21 network pairs were seen with subject age, based on correlation of age to between-network correlation, in either the Down Syndrome or control cohorts. p-Values for correlation between age and internetwork synchrony ranged from 0.2 to 0.8 in both cohorts. No other covariates were examined for correlation with imaging results.

### 3.2 Spatial distribution of connectivity differences

**Fig. 4:**
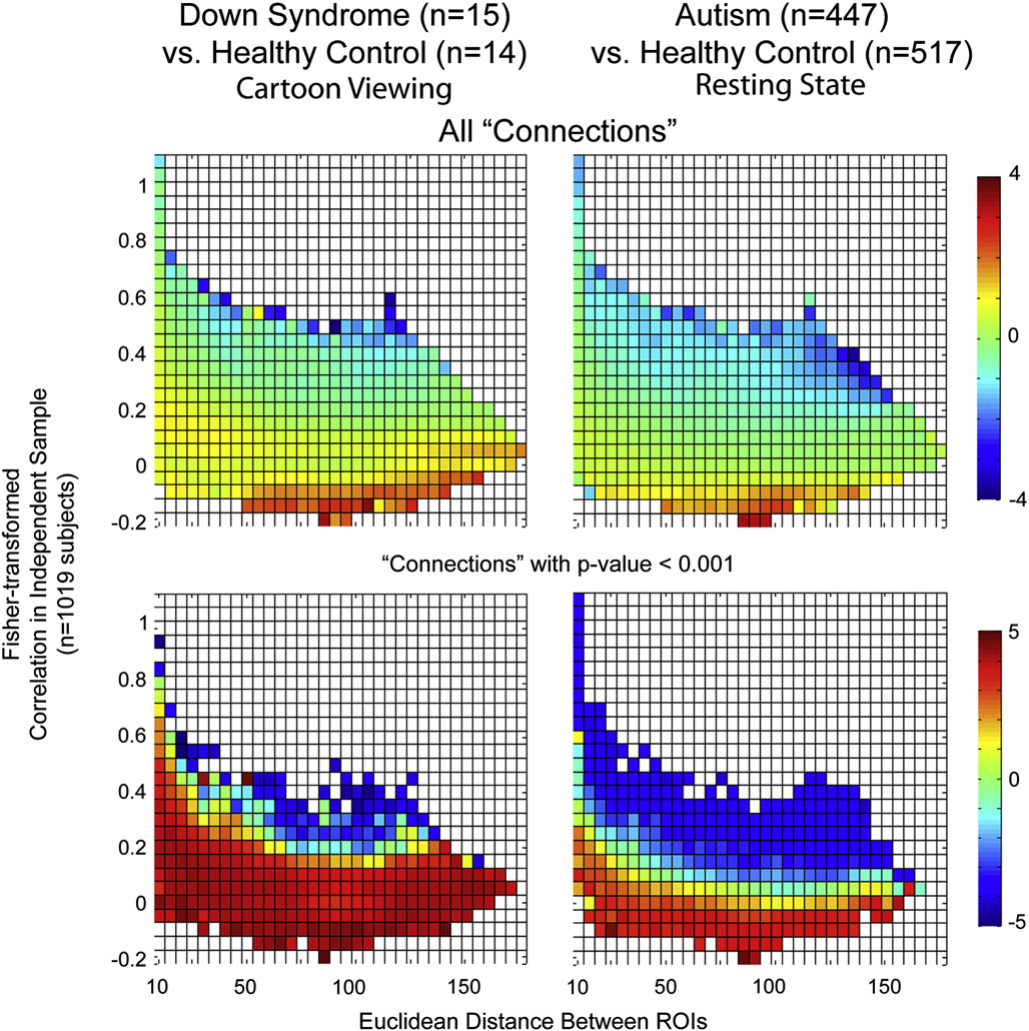
Intergroup differences in functional connectivity with respect to Euclidean distance and correlation strength of connections. 26.3 million “connections” were grouped into bins based on distance between ROIs in mm and mean correlation for the connection in a sample of 1019 subjects from the 1000 Functional Connectome and ADHD 200 datasets. Within each bin, the mean T-score (two-tailed *t*-test) between Down Syndrome and control groups (left) or autism and control groups (right) is shown. Above; Mean T-score between groups for all “connections” in the bin. Below; Mean T-score between groups for all “connections” in the bin with p-value < 0.001.

To assess in more detail the spatial distribution of connectivity differences in Down Syndrome, we obtained time series for each of 7266 regions of interest (ROIs) covering the gray matter at 5 mm spatial resolution (Anderson et al., 2011b, 2011d) and calculated pairwise Fisher-transformed correlations between each pair of ROI's in each subject. Overall trends with respect to connectivity differences associated with distance between regions and correlation strength in a large independent sample of control subjects (Ferguson and Anderson, 2011) are shown in Fig. 4 between control and Down Syndrome groups. For comparison, analogous results are shown from a sample of 964 subjects (447 autism, 517 healthy control) from the ABIDE resting state fMRI dataset. In the graphs above in Fig. 3, blue bins show sets of “connections” for which mean correlation was higher in control subjects, while red bins show sets of “connections” for which mean correlation was higher in patient groups (Down Syndrome or autism). All region pairs are included in the graphs above, and only region pairs for which patient/control p-values in a two-tailed *t*-test were less than p < 0.001 are included in the lower set of graphs.

A correlation measurement between two ROI's does not necessarily indicate that the two ROI's are linked by a structural connection, since synchrony can also result from shared input from other brain regions. With this caveat, pairs of ROI's are termed “connections” in the analysis below. Connections were grouped into bins based on the Euclidean distance in mm between the ROIs and the mean correlation across 1019 healthy control subjects from the 1000 Functional Connectome and ADHD 200 datasets, obtained with identical preprocessing methodology (Ferguson and Anderson, 2011). This approach reduced the number of comparisons to be made and allowed evaluation for general trends in connectivity associated with short vs. long-range connections and anticorrelated vs. strongly correlated connections. Bins were determined from resting state acquisitions, while Down Syndrome vs. control measurements were obtained from data during cartoon viewing. Thus, it is likely that systematic differences in functional connectivity are present during cartoon viewing, with a null hypothesis that these would affect Down Syndrome and control subjects similarly. There is possible overlap in control subjects from ABIDE and ADHD 200 datasets, and bin assignments were calculated both with and without the ADHD 200 subjects, with minimal differences that did not impact the results of Fig. 4.

Compared to controls, the Down Syndrome sample exhibited three main differences. 1) Short range connections (between ROIs less than 4 cm distant) showed higher functional connectivity in Down Syndrome; 2) a small subset of very strong, longer-range connections (between 6 and 12 cm distant ROIs) were weaker in Down Syndrome than controls; and 3) anticorrelated ROIs showed less anticorrelation in the Down Syndrome sample than controls.

A similar distribution of abnormal connectivity was observed in autism subjects, obtained from 964 subjects from the ABIDE dataset with identical preprocessing steps, with a few notable differences. These data were all obtained in a resting state, no-task paradigm. These findings are strongly consistent with results previously reported in a much smaller, single-site dataset (Anderson et al., 2011d). In autism, bins of connections with positive correlation in the FCON 1000 and ADHD 200 datasets were less correlated, and anticorrelated connections were less anticorrelated. Increased correlation observed in Down Syndrome subjects among connections that were relative short range (less than 5 cm distant) was not observed in autism.

**Fig. 5:**
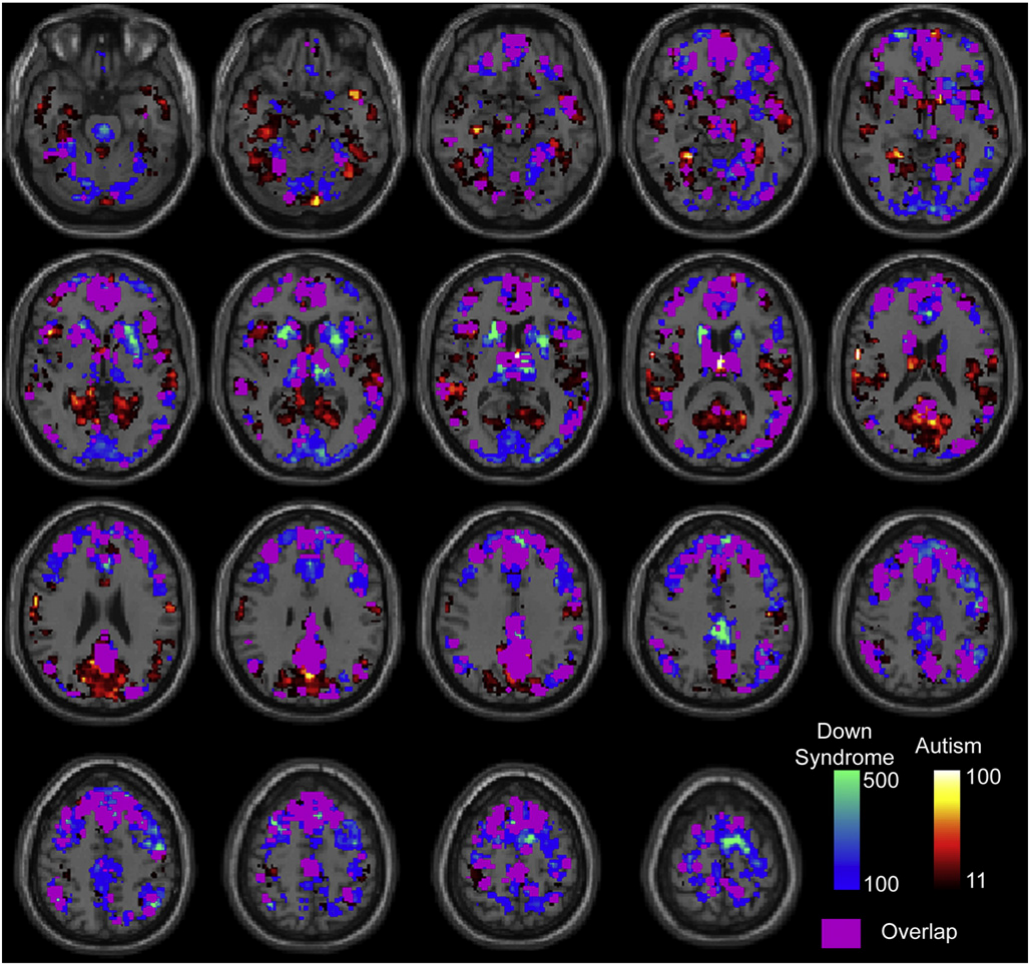
Spatial distribution of “connections” that differed between control and patient groups (at p-value < 0.001). Color scale shows the number of significant connections that had a given ROI as an endpoint. The top 50% of ROIs are shown in warm colors for autism and cool colors for Down Syndrome. ROIs common to both autism and Down Syndrome are colored in magenta. Images are in radiological format, with subject left on image right.

Brain regions most frequently involved in connections showing differences in Down Syndrome included medial prefrontal, anterior precuneus, intraparietal sulcus, head of caudate, putamen, and thalamus, with the greatest overlap between autism and Down Syndrome results seen in default mode network regions. Abnormal connections more frequently involve prefrontal cortex in Down Syndrome, with comparatively more involvement of temporal, parietal, and insular regions in autism. The spatial distribution of the most abnormal connections is shown in Fig. 5 for both Down Syndrome and autism samples, with the caveat that Down Syndrome results were obtained during cartoon viewing and autism results were obtained during a resting state.

### 3.3 Network community architecture

We next performed a graph-theoretical analysis of brain networks using the same 7266 nodes. A graph was obtained for each subject where an edge was included between two nodes if the Fisher-transformed correlation between the nodes was greater than either 0.1 (low-threshold condition) or 0.5 (high-threshold condition). Clustering coefficient, a measure of the proportion of edges containing a node out of all possible edges containing the node, was obtained for each node. Mean clustering coefficient across nodes was higher in Down Syndrome than for controls (low threshold condition: DS 0.636 +/− 0.024, HC 0.543 +/− 0.019, p = 0.007; high threshold condition: DS 0.344 +/− 0.013, HC 0.277 +/− 0.014, p = 0.002).

**Fig. 6:**
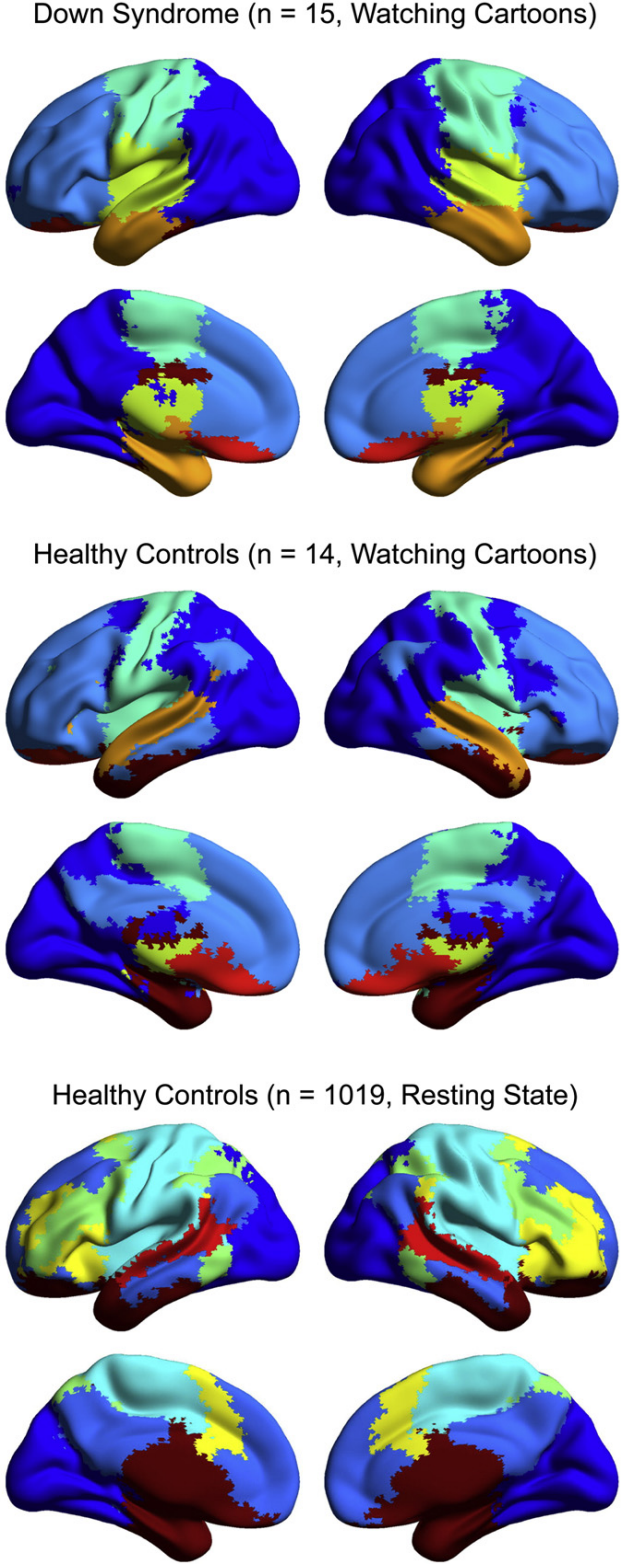
Parcellation of the brain into functional communities for Down Syndrome, healthy control, and large control samples. The Down Syndrome and healthy control data were obtained during cartoon viewing, while the large control sample was obtained from shorter epochs of resting state data. Each of the images was thresholded at 7 networks. Therefore, the red/brown regions of each image are in fact compilations of multiple networks.

A 7266 × 7266 correlation matrix was obtained for each subject, then averaged across all Down Syndrome and all control subjects separately to obtain a mean association matrix for each condition during the cartoon viewing task. A similar matrix was constructed from 1019 control subjects from the FCON 1000 and ADHD 200 datasets acquired during a resting state condition. A parcellation of the brain was performed using the Infomap algorithm (Power et al., 2011; Rosvall and Bergstrom, 2008) for each subject population. The 7 most populous communities (comprising almost all of the brain) are shown for each of the three samples in Fig. 6. Although there are differences between the two control samples, the overall community structures are similar. The visual and dorsal attention networks lie in a single community in the smaller control sample, possibly because the data was obtained during cartoon watching when visual and attentive regions may both show synchronization to cartoon stimuli. The medial prefrontal, posterior cingulate, and temporoparietal junction hubs of the default mode network are in the same community in both samples. The ventral attention network was not resolved in the smaller control sample. But in the Down Syndrome case, the network structure is vastly simplified, with communities defined by local proximity. The posterior hubs of the default mode network (i.e., temporoparietal junction, posterior cingulate cortex, and lateral temporal cortex) are not seen, and the attention control network is not resolved as a cluster.

### 3.4 Intersubject synchronization

**Fig. 7:**
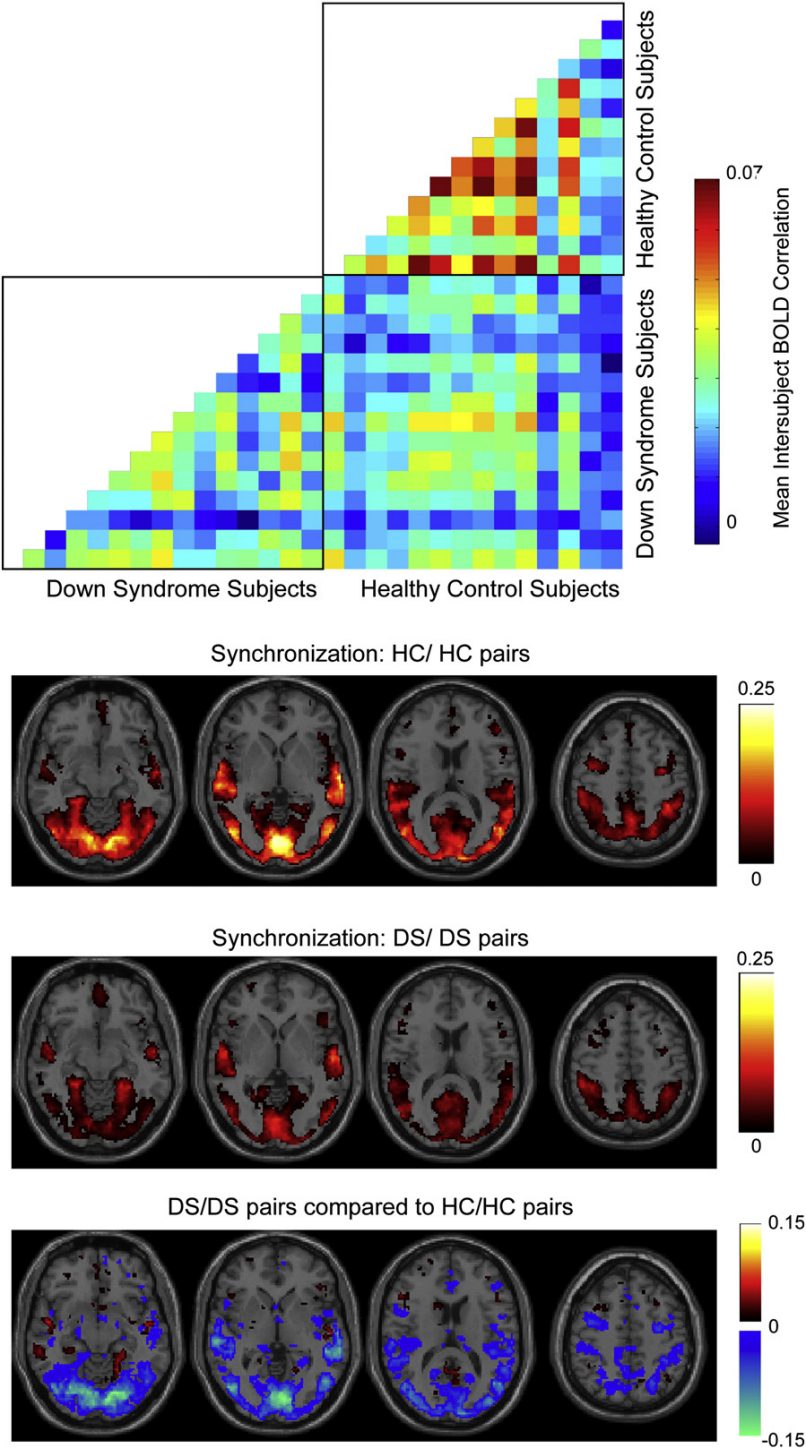
Intersubject synchronization to cartoon stimuli. Top; Intersubject synchronization between each pair of subjects. The entire 50 minute time series was correlated in each of 7266 ROIs, and Fisher-transformed intersubject correlation was averaged across all ROIs for each subject pair, with the result shown in color in the plot. Below; Significant intersubject correlation among control/control and DS/DS subject pairs as well as for significant differences between the two sets of subject pairs, determined by a Wilcoxon rank sum test. An acceptable False Discovery Rate (FDR, q < 0.05) was allowed for the set of all p-values for intersubject synchronization measurements from all regions and all subject pairs. The minimum correlation associated with p-values less than this threshold was chosen as a significant correlation threshold, and regions for which mean correlation across subject pairs was greater than this correlation value were deemed significant and shown in color on the images. Color scale shows mean Fisher-transformed correlation across all subject pairs for a given region.

Because all subjects were imaged while watching the same ten five-minute cartoons in the same order, with onset of the stimuli precisely timed to the onset of the scans, we were able to make additional comparisons about the relative timing of brain activation to stimuli between groups. We measured the time series across all ten cartoons for each of the same 7266 regions used in the connectivity analysis, and measured synchronization across pairs of subjects. Since the only thing in common between two different subjects is the stimuli, brain regions that show synchronization across subjects are likely engaged in processing the stimuli (Hasson et al., 2004, 2008). In Fig. 7, we show brain regions that were significantly synchronized across 91 pairs of 14 control subjects and in 105 pairs of 15 Down Syndrome subjects. Synchrony was evident in the primary and secondary visual cortices, Wernicke Area and right-sided homologue (receptive language), and attentional hubs (intraparietal sulcus, middle temporal, frontal eye fields). Significant synchronization was determined by a Wilcoxon rank sum test across subject pairs for correlation of the same ROI in both subjects, compared to correlation of scrambled versions of the same time series, corrected for multiple comparisons using q < 0.05 False Discovery Rate across ROIs.

Down Syndrome subjects were less synchronized to other Down Syndrome or control subjects than were control subjects to other control subjects. Thus, even though connectivity analyses showed greater synchrony of brain networks in Down Syndrome, this did not translate to more reproducible responses to stimuli across subjects. In contrast, these data indicate that brain responses to stimuli are less robust and more idiosyncratic in Down Syndrome subjects.

## 4 Discussion

BOLD fMRI images were obtained for cohorts of Down Syndrome and typically developing control subjects while watching video cartoon stimuli. Down Syndrome subjects showed higher levels of synchrony between distributed brain networks as well as between the vast majority of gray matter regions. Among longer-range connections between brain regions greater than 6 cm apart, Down Syndrome subjects exhibited weaker correlation only for a relatively small subset of the most correlated regions, whether negatively or positively related. Regardless of the distance separating regions, pairs of regions that showed anticorrelation in a large control sample showed increased correlation (reduced anticorrelation) in Down Syndrome. A large sample of autism and control subjects showed similar differences, but to a smaller degree, and autism subjects did not exhibit the shorter-range increased synchrony seen in Down Syndrome. This generalized increase in brain synchrony did not equate to more uniform or robust response to video stimuli. In contrast, temporal brain activity patterns of Down Syndrome individuals were less correlated to those of either other Down Syndrome or control subjects than were control subjects.

Down Syndrome and autism cohorts also differed in between-network correlation. Down Syndrome subjects showed increased synchrony between distributed networks for all network pairs, whereas autism subjects showed increased synchrony only between the default mode network and networks processing attention to external stimuli such as the ventral attention network and frontoparietal attention network. Although intersite variability in the ABIDE dataset may limit sensitivity, the abnormalities were much more similar to controls than for Down Syndrome. A previous analysis of ABIDE data showed many sources of variability between sites, but agreement on core findings of predominantly hypoconnectivity for interhemispheric and cortico-cortical connectivity (DiMartino et al., 2013). Our results extend these findings by showing that between network connectivity is most abnormal between the brain's default mode network and brain attentional networks, and that negatively correlated connections are weaker in autism. That between-network connectivity abnormalities were limited to internal vs. external attentional networks is intriguing, given the well characterized abnormalities of default mode network connectivity in autism (Assaf et al., 2010; Kana et al., 2009; Kennedy and Courchesne, 2008; Lombardo et al., 2010; Mason et al., 2008; Monk et al., 2009; Weng et al., 2009), and the failure of default mode network deactivation during attentional tasks (Kennedy et al., 2006). Given the reported anticorrelation between default mode and attentional networks (Fox et al., 2005), these findings may imply that weaker segregation of intrinsic internal and external attentional networks is a specific marker of connectivity abnormalities in autism.

These findings provide a first assessment of patterns of abnormal brain network connectivity in Down Syndrome, and represent one of the first characterizations of functional connectivity in a severely low-functioning population. Prior structural imaging and clinical findings in Down Syndrome have identified abnormalities in cerebral volumes of the hippocampi (Pinter et al., 2001), cingulate gyrus (White et al., 2003), and temporal lobes (White et al., 2003), as well as the cerebellar vermis where volume reductions have been associated with impaired gait (Rigoldi et al., 2009). We also find that the cingulate gyrus appears overrepresented among connections that showed significantly different functional connectivity between groups (Fig. 5), although the most commonly involved regions were the bilateral head of caudate, putamen, and thalami in our results. These findings have not only similarities but also differences with areas of greatest abnormalities in brain connectivity in autism, where association cortex regions in the default mode and attention control networks show the greatest involvement (Anderson et al., 2011d), with particular involvement of regions comprising social brain function (Gotts et al., 2012). Corticostriatal connectivity has also been found to be abnormal in autism, with increased connectivity in corticostriatal projections (Di Martino et al., 2011).

The distribution of functional connectivity abnormalities also correlates with behavioral studies of abnormalities in Down Syndrome. The predominance of connectivity disturbances in prefrontal cortex is in agreement with results from the Arizona Cognitive Test Battery, specifically designed for Down Syndrome, where prefrotal neuropsychological measures are among the most salient abnormalities seen in Down Syndrome (Edgin et al., 2010). Measures of response inhibition were also strongly abnormal, with metrics of inhibition correlated with parental report scores and function (Edgin et al., 2010). Nevertheless, inhibition in a neuropsychological testing construct may be indirectly or unrelated to widespread abnormalities in negative correlations we observe in functional connectivity.

Abnormal brain synchrony in Down Syndrome is reflected in a simplified pattern of distributed brain networks with respect to the control subjects in our sample. Some differences may be related to acquisition using cartoon video stimuli, but both control groups exhibit canonical functional connectivity networks with distributed hubs, whereas the Down Syndrome network organization is characterized by a lobar architecture dominated by local connectivity relationships. This may be a consequence of weak or absent negative correlations between brain regions as well as decrease in strong, long-distance correlations. If these communities represent functional domains within the brain, it would suggest an impaired ability in Down Syndrome to aggregate information from distant brain regions into coherent networks, with decreased specialization and segregation of large-scale association cortex networks (Fair et al., 2007).

Although Down Syndrome subjects exhibited more globally synchronized brain networks, this synchrony was not adaptive in the sense of responding to external stimuli. Despite *increased* internetwork synchrony, Down Syndrome subjects showed *decreased* intersubject synchronization in response to the audiovisual content of the cartoons. Thus, the intrinsic brain activity measured was more idiosyncratic with less evidence of processing content of the cartoons in distributed brain networks in Down Syndrome. This was observed within Down Syndrome subjects, which were not well synchronized with each other in response to the cartoons, as well as when compared to control subjects, with weaker synchronization to control subjects than were other control subjects to each other.

Our findings do not directly support a prevailing hypothesis of neuropathology in Down Syndrome of increased inhibition/excitation ratios. A mouse model for trisomy 21, Ts65Dn, exhibits impaired long-term potentiation due to reduced activation of NMDA receptors, with rescue of LTP when inhibition is suppressed by a GABA_A_ antagonist (Belichenko et al., 2004; Kleschevnikov et al., 2004). Yet it is difficult to directly compare our results to electrophysiologic findings in the mouse model because there may be divergent findings for acute vs. chronic effects, and because we are accruing data from large network regions rather than microcircuitry. At least one study has found hyperconnectivity in Ts65Dn mice in the CA3 region of the hippocampus (Hanson et al., 2007). We did not analyze brain connections specific to the hippocampus and do not have sufficient spatial resolution to inform a discussion of hippocampal microcircuitry, but if therapeutic treatment of Down Syndrome is approached by curbing inhibition in corticocortical circuits, it would be helpful to know the extent to which local connectivity increases we observe may be caused by impaired cortico-cortical inhibitory connections within the neocortex. Our results could be consistent with impaired long-range inhibition, to the extent that negatively correlated brain regions reflect on some level inhibitory projections as opposed to withdrawal of excitation. A summary of reported findings is included in Table 2, below.

Our data might be consistent with a hypothesis of impaired inhibitory circuitry in Down Syndrome, which might be manifested by a reduction in anticorrelation of brain regions, decreased differentiation of brain regions, and increased global synchrony. Such a hypothesis might also be consistent with the increased prevalence of epilepsy in Down Syndrome (McVicker et al., 1994). Nevertheless, impaired inhibition is only one possible mechanism for functional connectivity findings we observe. In particular, reduced anticorrelation among brain regions may be due to decreased inhibition, withdrawal of excitation, or complex network effects of shared inputs, and a clear interpretation of these findings will require corroborating evidence from other methodologies in both human and animal models such as direct electrophysiologic measurements.

Generalized increases in connectivity in Down Syndrome, despite provocative implications for neuropathological mechanisms of intellectual disability, are preliminary. It has been reported that greater subject motion during scanning can result in artifactual higher local connectivity and reduced long-range connectivity (Power et al., 2012), which is precisely what we observe. Moreover, it has been consistently reported that the brains of Down Syndrome individuals show reduced intracranial volume relative to typically developing individuals (Aylward et al., 1999; Kesslak et al., 1994; Raz et al., 1995). A consequence of this fact might be that when normalization to MNI space is performed, each original voxel during imaging acquisition will be volume averaged with neighboring voxels since a net expansion will occur during normalization. Nevertheless, increased connectivity is also observed many centimeters away and between distributed brain networks where volume averaging would be an unlikely factor.

We took care to perform rigorous corrections for subject motion including initial motion correction, regression of motion parameters, and scrubbing of all frames where subjects exhibited significant motion. Even more rigorous scrubbing of the Down Syndrome group appeared to have no effect on our results. The experimental design also helped mitigate the effects of subject motion by acquiring long imaging times per subject and allowing exclusion of frames with subject motion while retaining large numbers of volumes per subject for analysis. Further studies, perhaps involving electrophysiological methods or imaging techniques where normalization is not required, could help confirm the extent to which local connectivity increases in Down Syndrome is reproducibly present. Our data provide an additional hypothesis that could be tested with electrophysiological techniques of delayed temporal activation patterns to ongoing natural stimulation.

The age range for the Down Syndrome and control cohorts is relatively broad for the present study, ranging from adolescence through adulthood. A modest sample size limits the opportunity to probe differences in connectivity in Down Syndrome associated with age throughout development. No difference in internetwork correlation was seen associated with age in either the Down Syndrome or control cohort for any of the 21 network pairs in our sample. Nevertheless, developmental differences in connectivity are not excluded by our study and will require additional study.

In order to facilitate longer scan times with a more homogenous brain state, we acquired images during cartoon viewing. Given that auditory and visual inputs were present during the acquisition, there are likely to be systematic changes in functional connectivity associated with synchronization to audiovisual inputs, as well as synchronized co-activation of higher order attentional and language regions. We exploit these differences when evaluating intersubject synchronization. Nevertheless some care is required in directly comparing resting and task acquisitions, given the likelihood of systematic connectivity differences. In a prior report, these differences were substantive enough that a classifier could distinguish resting and cartoon acquisitions based on functional connectivity with as little as five minutes of imaging time (Anderson et al., 2011c). We acknowledge that Down Syndrome subjects may differentially respond to the content of the cartoons in ways that affect connectivity results described above, although this possibility would also not be excluded during a resting acquisition given that a “resting state” is itself a task that can be differentially performed, albeit less constrained and amenable to characterization.

Even in a modest sample of 15 Down Syndrome subjects, significant abnormalities in connectivity are readily apparent. Given that Down Syndrome subjects are lower-functioning than most other clinical populations studied, it is possible that connectivity abnormalities we observe are nonspecific to Down Syndrome but are downstream consequences of the genetic abnormalities that are correlated with and/or responsible for the severity of impaired cognitive function. The inverse relationship between performance IQ and brain synchrony suggests that abnormal brain synchrony is not an artifact of head motion, respiration, or vascular etiology, and studies in other low-functioning populations could assess whether increased brain synchrony is specific to the pathophysiology of Down Syndrome.

## Figures and Tables

**Table 1:** Down Syndrome, autism, and control subjects included in the analysis. Age, verbal IQ, and performance IQ mean and standard deviation are reported for each sample. Age range is shown in parenthesis.

	Age	VIQ	PIQ
Down Syndrome (n)	15 (9 M, 6 F)	11	11
DS mean +/− s.d.	20.2 +/− 6.3 (14–34)	49.2 +/− 12.1	46.2 +/− 5.7
Control (n)	11 (8 M, 6 F)	6	6
Control mean +/− s.d.	23.7 +/− 5.9 (15–39)	110.2 +/− 10.8	111.7 +/− 16.2
p-Value (two-tailed *t*-test)	0.14	4.8 ∗ 10^− 8^	2.6 ∗ 10^− 9^
Control (resting state)	1019	296	296
FCON 1000	(374 M, 249 F)		
ADHD 200	(216 M, 180 F)		
Control mean +/− s.d.	18.3 +/− 5.6 (7–30)	114.9 +/− 13.7	110.2 +/− 14.0
ABIDE (resting state)	964	781	796
Control	(426 M, 91 F)	413	425
Autism	(396 M, 51 F)	368	371
Control mean +/− s.d.	16.9 +/− 7.6 (6.5–56.2)	111.6 +/− 13.3	108.2 +/− 13.3
Autism mean +/− s.d.	16.6 +/− 8.1 (7.0–64.0)	104.8 +/− 17.8	105.6 +/− 17.2

**Table 2:** Reported findings in the present study and in the Ts65Dn mouse model of Down Syndrome.

Functional connectivity MRI findings (human)	Ts65Dn mouse model
Weak long-range positive connections	Reduced activation of NMDA receptors
Weak negative connections (all distances)	Impaired LTP
Increased short range (1–5 cm) connections	Increased inhibition/excitation ratio
Simplified network architecture	LTP rescued by GABA_A_ antagonist
	Hyperconnectivity of hippocampal CA3
